# Therapeutic Potential of Mangosteen Pericarp Extract-Loaded Liposomes against Superficial Skin Infection Caused by *Staphylococcus pseudintermedius* in a Murine Model

**DOI:** 10.3390/antibiotics13070612

**Published:** 2024-07-01

**Authors:** Seong-Yeop Kim, Seong-Yong Park, Jung-Hwa Lee, Nayeong Kim, Ha-Na Oh, So-Young Yoo, Dae-Sung Lee, Je-Chul Lee

**Affiliations:** 1 Department of Microbiology, School of Medicine, Kyungpook National University, Daegu 41944, Republic of Korea; dgsk0207@naver.com (S.-Y.K.); psyseongyong@knu.ac.kr (S.-Y.P.); biotech77@hanmail.net (J.-H.L.); tbc02021@knu.ac.kr (N.K.); 2 Medi Bio Lab Co., Ltd., Seoul 08389, Republic of Korea; jhs2hn@naver.com (H.-N.O.); smileyoo1@naver.com (S.-Y.Y.); dsl2008@naver.com (D.-S.L.); 3 Untreatable Infectious Disease Institute, Kyungpook National University, Daegu 41944, Republic of Korea

**Keywords:** mangosteen pericarp, α-mangostin, *Staphylococcus* species, superficial pyoderma, companion animals

## Abstract

α-mangostin (α-MG) demonstrates antibacterial activity against *Staphylococcus* species. Therefore, this study aimed to explore the antibacterial activity of α-MG-rich mangosteen pericarp extract (MPE)-loaded liposomes against *Staphylococcus* isolates from companion animal skin diseases in vitro and evaluated their therapeutic potential in a murine model of superficial skin infection caused by *S. pseudintermedius*. α-MG-rich extract was purified from mangosteen pericarp and then complexed with γ-cyclodextrin (γ-CD), forming the inclusion complexes. Nanoliposomes containing MPE and γ-CD complexes were prepared by adding lecithin and casein. Minimum inhibitory concentrations (MICs) and minimum bactericidal concentrations (MBCs) of MPE-loaded liposomes were determined using agar dilution and broth microdilution methods. The therapeutic potential of MPE-loaded liposomes was evaluated in vivo on tape-stripped skin lesions infected with *S. pseudintermedius*. Purified MPE and MPE-loaded liposomes contained 402.43 mg/g and 18.18 mg/g α-MG, respectively. MPE-loaded liposomes showed antibacterial activity against clinical *Staphylococcus* isolates in vitro but did not show antibacterial activity against Gram-negative bacterial isolates. MPE-loaded liposomes demonstrated consistent MICs and MBCs against *Staphylococcus* isolates. These liposomes significantly reduced bacterial numbers and lesional sizes in a superficial skin infection model. Moreover, they reconstructed the epidermal barrier in skin lesions. The therapeutic concentrations of MPE-loaded liposomes did not induce cytotoxicity in canine progenitor epidermal keratinocyte cells. In conclusion, MPE-loaded liposomes hold promise for the development of a prospective topical formulation to treat superficial pyoderma in companion animals.

## 1. Introduction

Superficial bacterial folliculitis, also called superficial pyoderma, is one of the most common causes of antimicrobial treatment in companion animal medicine [1]. *Staphylococcus pseudintermedius* is the primary causative agent of superficial pyoderma in companion dogs, followed by *S. schleiferi* and *S. felis* [2,3,4]. Topical antimicrobial therapy is a primary treatment choice for superficial pyoderma caused by *Staphylococcus* species [2]. However, antimicrobial resistance to commonly used antibiotics such as methicillin is increasingly prevalent in *Staphylococcus* species isolated from companion animals [5,6,7]. Therefore, this necessitates the development of antibiotic alternatives for superficial pyoderma in these animals.

Mangosteen (*Garcinia mangostana* Linn), also called queen of fruits and mangostana, has a long history of cultivation in Southeast Asia. Its pericarp has been used in traditional medicine for treating bacterial infections, such as wound and skin infections, diarrhea, and dysentery [8,9]. The primary bioactive compounds in mangosteen are xanthone derivatives, with α-mangostin (α-MG) exhibiting potent antibacterial activity against Gram-positive bacteria, including *Staphylococcus* species, *Streptococcus pyogenes*, and *Enterococcus* species [10,11,12,13,14]. α-MG binds to bacterial cell membranes, inducing membrane disruption and subsequent bacterial death [15]. α-MG exhibits antibacterial efficacy against clinical *Staphylococcus* isolates from companion dogs and cats in vitro [16]. Moreover, in an in vivo murine skin infection model induced by *S. pseudintermedius*, α-MG significantly decreases bacterial count and suppresses the inflammatory response in skin lesions. These finding suggest that α-MG-rich mangosteen pericarp extract (MPE) holds potential for the development of topical therapeutics for superficial pyoderma in companion animals.

However, the bioavailability of α-MG is constrained by its low water solubility (2.03 × 10^−4^ mg/L at 25 °C) and susceptibility to degradation under acidic hydrolytic conditions [17,18]. Water solubility is crucial for increasing drug bioavailability. Consequently, health products containing α-MG necessitate a high concentration of solubilizers, such as alcohol and surfactants, to improve solubility [19,20]. To develop an effective topical formulation of α-MG, encapsulation into nanoemulsions has been employed to enhance its water solubility, stability, and controlled release within tissues [20,21]. γ-cyclodextrin (γ-CD) serves as a complexing agent to enhance the solubility and stability of drugs. Inclusion complexes of α-MG and γ-CD increase the water solubility of α-MG to 31.7-fold [21]. Additionally, nanoparticle formulations such as nanoliposomes aid in stabilizing hydrophobic drugs such as α-MG [17]. Lecithin-based liposomes are a potent drug delivery system due to its physicochemical properties, such as self-assembly, large aqueous center, and high loading capacity [22]. Addition of casein in lecithin-based liposomes reduces the vesicle size, prolongs liposome lifespan, and enhances the stability of the hydrophobic drugs [23].

In this study, α-MG-rich extract was purified from mangosteen pericarp, which was then complexed with γ-CD. Subsequently, lecithin-based nanoliposomes containing the inclusion complexes of MPE and γ-CD were prepared by adding casein. The antibacterial activity of these MPE-loaded liposomes was evaluated against clinical *Staphylococcus* isolates and two Gram-negative bacterial species from skin diseases of companion dogs and cats in vitro. Furthermore, the therapeutic potential of MPE-loaded liposomes was evaluated in a murine skin lesion infected with *S. pseudintermedius*.

## 2. Results

### 2.1. Characterization of MPE-Loaded Liposomes

**Figure 1:**
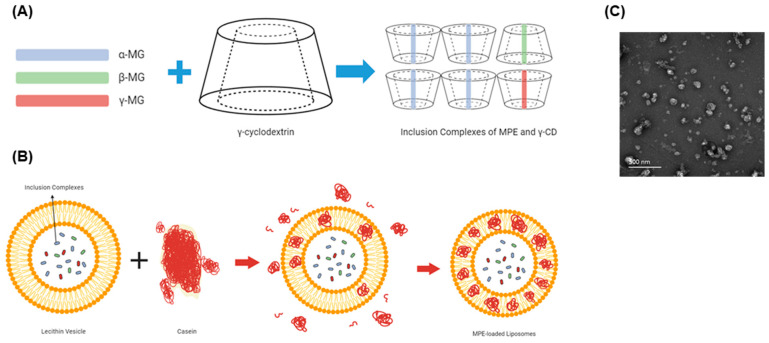
Preparation of mangosteen pericarp extract (MPE)-loaded liposomes. (**A**,**B**) Schematic representation of nanoliposomes. (**A**) Inclusion complexes of MPE and γ-cyclodextrin (γ-CD). (**B**) Lecithin-based nanoliposomes carrying the inclusion complexes of MPE and γ-CD by adding casein. (**C**) Transmission electron microscopic image of MPE-loaded liposomes. The scale bar represents 500 nm.

High-performance liquid chromatography (HPLC) analysis revealed that MPE contained 402.43 mg/g α-MG. This extract was complexed with γ-CD, forming inclusion complexes of MPE and γ-CD (Figure 1A). Nanoliposomes carrying these inclusion complexes were prepared from lecithin-based vesicles by adding casein (Figure 1B). These MPE-loaded liposomes were spherical particles (Figure 1C) and contained 18.18 mg/g α-MG.

### 2.2. Antibacterial Activity of MPE-Loaded Liposomes against Reference Bacterial Strains In Vitro

The minimum inhibitory concentrations (MICs) of MPE-loaded liposomes were determined against reference strains of *Staphylococcus* species and Gram-negative bacteria using agar dilution and broth microdilution methods. The MICs of MPE-loaded liposomes against various *Staphylococcus* species ranged from 55–220 µg/mL (1–4 µg/mL of α-MG concentrations) in the broth microdilution method and 55–880 µg/mL (1–16 µg/mL of α-MG concentrations) in the agar dilution method (Table 1). Among the tested *Staphylococcus* species, three common pathogens of skin disease in companion animals, *S. pseudintermedius*, *S. felis*, and *S. schleiferi*, exhibited the lowest MICs (55 µg/mL) in the broth dilution method. However, *S. aureus* displayed the highest MIC (220 µg/mL) in the broth microdilution method. All tested *Staphylococcus* species showed the same MIC and minimum bactericidal concentration (MBC) values. The MICs of MPE-loaded liposomes against two Gram-negative bacterial strains, *Escherichia coli* ATCC 25922 and *Pseudomonas aeruginosa* ATCC 27853, exceeded 3520 µg/mL (>64 µg/mL of α-MG concentrations), indicating negligible or weak antibacterial activity. These findings suggest that MPE-loaded liposomes demonstrate antibacterial activity against *Staphylococcus* species in vitro.

### 2.3. Antibacterial Activity of MPE-Loaded Liposomes against Clinical Isolates In Vitro

The MICs of MPE-loaded liposomes were determined against 241 clinical isolates from skin diseases of companion dogs and cats using the agar dilution method. The MICs of MPE-loaded liposomes against all *Staphylococcus* isolates ranged from 28–880 µg/mL (0.5–16 µg/mL of α-MG concentrations) (Table 2). The MIC_50_ and MIC_90_ of MPE-loaded liposomes were lowest (110 µg/mL) in *S. pseudintermedius* isolates, but highest (880 µg/mL) in *S. aureus* isolates. For 36 clinical Gram-negative bacterial isolates, MICs of MPE-loaded liposomes exceeded 3520 µg/mL (>64 µg/mL of α-MG concentrations). These findings suggest that MPE-loaded liposomes possess potent antibacterial activity against three common *Staphylococcus* species associated with skin diseases of companion animals in vitro.

### 2.4. Host Cell Cytotoxicity of MPE-Loaded Liposomes In Vitro

**Figure 2:**
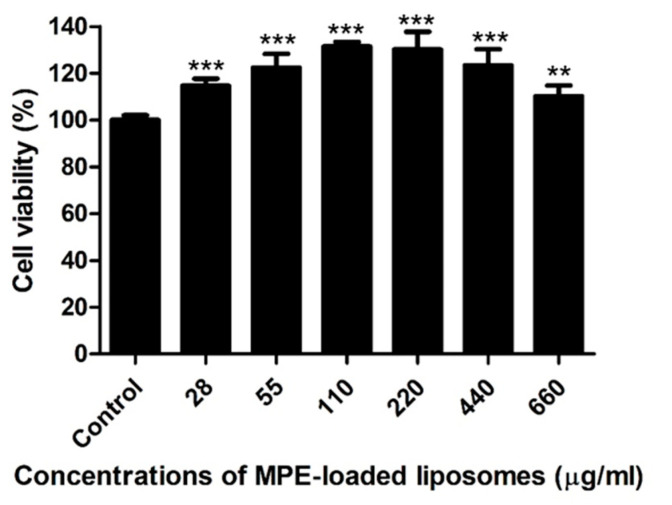
Viability of canine progenitor epidermal keratinocytes treated with mangosteen pericarp extract (MPE)-loaded liposomes. Cells were exposed to various concentrations of MPE-loaded liposomes for 24 h, and MTT assays were performed. Data are expressed as mean ± SD of three independent experiments. ** *p* < 0.01 and *** *p* < 0.001 (Student’s *t*-test) compared with the cells treated with 1% DMSO.

Canine progenitor epidermal keratinocyte (CPEK) cells were exposed to various concentrations (14–660 µg/mL) of MPE-loaded liposomes for 24 h. As a control, cells were treated with 1% dimethyl sulfoxide (DMSO), the solvent for MPE-loaded liposomes. MPE-loaded liposomes did not induce cytotoxicity at concentrations ≤660 µg/mL (≤12 µg/mL of α-MG concentrations), but significantly induced the proliferation of CPEK cells (Figure 2).

### 2.5. Therapeutic Potential of MPE-Loaded Liposomes in a Murine Superficial Skin Infection Model Caused by S. pseudintermedius

**Figure 3:**
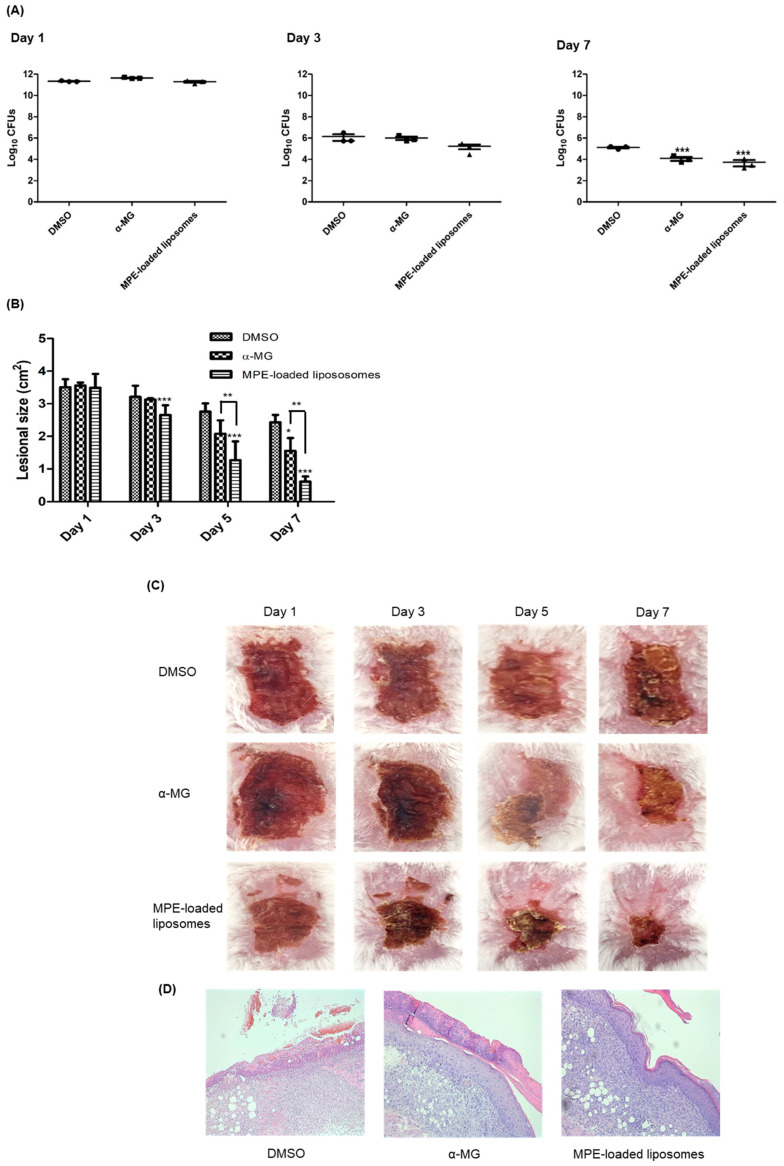
In vivo effects of mangosteen pericarp extract (MPE)-loaded liposomes on tape-stripped skin lesions infected with *S. pseudintermedius*. *S. pseudintermedius* ATCC 49051 (1.0 × 10^9^ CFUs/100 µL) was inoculated onto tape-stripped skin lesions. Treatment with α-mangostin (α-MG, 10 µg), MPE-loaded liposomes (550 µg, 10 µg in α-MG concentrations), and 1% DMSO in PBS (control) were applied topically to skin lesions every 12 h for 6 days. The CFUs (**A**), skin lesion size (**B**), regional skin lesions (**C**), and histological changes (**D**) examined 6 h after the last treatment. The samples were stained with hematoxylin and eosin (H&E) (**D**) and viewed at 200× magnification. * *p* < 0.05, ** *p* < 0.01, and *** *p* < 0.001 compared with control mice treated with 1% DMSO in PBS.

The therapeutic efficacy of MPE-loaded liposomes was assessed in a murine superficial skin infection model. *S. pseudintermedius* ATCC 49051 was inoculated into the tape-stripped skin lesions, and MPE-loaded liposomes were applied every 12 h for 6 days. α-MG and 1% DMSO in phosphate-buffered saline (PBS) served as positive and negative controls, respectively. On day 3, no significant differences were observed in the colony-forming units (CFUs) of skin lesions among the three groups. However, the CFUs in skin lesions treated with MPE-loaded liposomes exhibited a slight reduction to 8.3-fold (1.67 × 10^5^ CFUs) compared to those treated with 1% DMSO in PBS (1.40 × 10^6^ CFUs) (Figure 3A). The CFUs decreased significantly in mouse groups treated with α-MG and MPE-loaded liposomes at day 7. The reduction in CFUs was more pronounced in MPE-loaded liposome-treated groups than in the α-MG-treated group. Subsequently, to determine the wound healing potential of MPE-loaded liposomes in skin lesions, the size of skin lesions was measured. MPE-loaded liposomes significantly reduced lesional size on days 3, 5, and 7 (Figure 3B). Additionally, α-MG also significantly reduced the skin lesion size compared to the control mice treated with DMSO at day 7. Moreover, the reduction of lesion size was significantly different between α-MG and MPE-loaded liposomes on days 5 and 7. At day 7, control mice treated with DMSO exhibited reddish lesions covered with eschar, whereas mice treated with MPE-loaded liposomes showed normal skin appearance with small eschars (Figure 3C). In histological assessment at day 7, control mice treated with DMSO showed incomplete epidermal barrier in skin lesions (Figure 3D). However, treatment with α-MG and MPE-loaded liposomes exhibited reconstruction of epidermis. These findings suggest that MPE-loaded liposomes exhibit antimicrobial and wound healing effects in skin lesions infected with *S. pseudintermedius*.

## 3. Discussion

This study demonstrated that the novel formulation of MPE-loaded liposomes exhibited antibacterial activity against clinical *Staphylococcus* isolates from skin diseases of companion dogs and cats in vitro. Furthermore, in a murine superficial skin infection model induced by tape-stripping and *S. pseudintermedius* infection, topical application of MPE-loaded liposomes significantly decreased bacterial number and promoted wound healing effect in the skin lesions. These results indicate that MPE-loaded liposomes are a promising candidate to develop topical formulations for superficial pyoderma in companion animals.

The controlled cooling crystallization method employed in this study resulted in relatively higher α-MG concentrations in MPE (402.43 mg/g) compared to other α-MG extraction protocols such as ethyl acetate extraction and maceration and column chromatography [24,25,26]. This large scale preparation of α-MG-rich MPE is cost-effective for developing veterinary phytopharmaceutical products. γ-CD serves as a complexing agent in medicine, pharmacy, the food industry, and cosmetics. Its large cavity size thus accommodates large biomolecules [21,27]. Inclusion complexes of MPE and γ-CD were prepared to increase water solubility and prevent biodegradation [21]. Lecithin-based liposomes carrying these complexes were prepared by adding casein. This might be efficient in delivering α-MG-rich MPE in skin tissue and reducing the degradation of bioactive molecules within MPE [22,23]. In the prior study, α-MG in pluronic lecithin organogel (PLO) significantly reduced the CFUs and suppressed inflammation in murine skin lesions induced by *S. pseudintermedius*, indicating the effectiveness of PLO as a delivery vehicle for α-MG. However, large scale preparation of α-MG in PLO is complex and expensive. Therefore, a novel formulation of lecithin-based liposomes with casein was prepared in this study. The water solubility of MPE-loaded liposomes was dramatically improved to 7 mg/mL. Furthermore, MPE-loaded liposomes exhibited no increased cytotoxic activity against CPEK cells compared to α-MG alone. However, further investigation is essential to characterize physicochemical and biophysical properties of MPE-loaded liposomes, such as efficiency of inclusion complex and liposome formation, release of xanthones from liposomes, tissue penetration, and stability under harsh conditions. This is essential for the development of MPE-loaded liposomes as a topical therapeutics for superficial pyoderma in companion animals.

The antibacterial activity of MPE-loaded liposomes against reference strains of *Staphylococcus* species was evaluated using agar dilution and broth microdilution methods. In the broth microdilution method, the MICs of MPE-loaded liposomes against these strains ranged from 55–220 μg/mL, which can be translated to α-MG concentrations of 1–4 μg/mL. The MICs of MPE-loaded liposomes against *Staphylococcus* species reference strains were either the same or exhibited a ≤2-fold difference compared to the MICs of α-MG tested using the broth microdilution method [16]. The MICs and MBCs of α-MG differed 2- or 4-fold against all tested *Staphylococcus* species [16]. However, the MICs and MBCs of MPE-loaded liposomes the same against these strains. This suggests that α-MG in MPE is primarily responsible for antibacterial activity, but other bioactive ingredients, such as β- and γ-mangostin, also play a role in antibacterial activity. Next, we assessed the antibacterial activity of MPE-loaded liposomes against clinical isolates of *Staphylococcus* species, *E. coli*, and *P. aeruginosa*. Of the five tested *Staphylococcus* species, MPE-loaded liposomes displayed lower MICs against *S. pseudintermedius*, *S. schleiferi*, and *S. felis*, which were prevalent in skin diseases of companion animals, than *S. aureus* and *S. epidermidis*, which were primarily associated with human pathogens. However, MPE-loaded liposomes showed minimal or weak antibacterial activity against Gram-negative bacterial species, like α-MG [16].

Skin lesions on the back were treated with 550 µg of MPE-loaded liposomes (10 µg of α-MG concentrations) to determine therapeutic potential of liposome formulation of MPE in vivo. The MPE-loaded liposomes demonstrated robust therapeutic activity based on the reduction of bacterial number, decreased lesional size, and histological reconstruction of epidermal barrier. The wound healing effect was more pronounced with MPE-loaded liposomes than α-MG alone. Although this study did not investigate the immune response of MPE-loaded liposomes, α-MG, a major xanthone in the MPE-loaded liposomes, inhibited the expression of pro-inflammatory and Th1, Th2, and Th17 cytokine genes in vivo [16]. These results suggest that MPE-loaded liposomes also play a role in suppression of inflammatory response in a murine model. Similarly, nanoformuations of α-MG, including liposomes, have been developed for cancer drug delivery [28]. Additionally, α-MG proniosomes were developed to enhance the skin permeation of α-MG [29]. Proniosomes incorporating soya lecithin improved the water solubility of α-MG. However, liposomes carrying α-MG or MPE for antibacterial activity have not been reported yet. Limitation of this study was the application of MPE-loaded liposomes in a murine superficial skin infection model rather than a superficial pyoderma model in companion animals. Since *S. pseudintermedius* is a normal microbiota of dogs, the spread of *S. pseudintermedius* from normal skin to lesional skin and its effect on skin lesions could not be evaluated in this study.

## 4. Materials and Methods

### 4.1. Preparation of MPE

Mangosteen fruits were purchased from a local grocery store in Seoul, Korea. The pericarps were washed with tap water, chopped, and dried in an oven at 45 °C for 48 h. The dried pericarps were then ground into a fine powder (40 mesh). MPE was prepared using the controlled cooling crystallization method. In brief, the mangosteen pericarp powder was dissolved in 80% ethanol at 80 °C for 4 h in a crystallizer (Biocean, Seoul, Republic of Korea), cooled to 4 °C at a rate of −0.1 °C/min, and aged at 4 °C for 24 h. After filtration at 4 °C to remove small pieces and debris, the solid phase extract was recovered and dried using a spray dryer (Mehyun Engineering Co., Anyang, Republic of Korea) for 24 h. The spray drying conditions were 180 °C, 90 °C, and 120 bar for inlet temperature, outlet temperature, and nozzle pressure, respectively.

### 4.2. Preparation of Inclusion Complex of MPE and γ-CD and MPE-Loaded Liposomes

The prepared MPE was dissolved in 95% ethanol at 50 °C for 30 min and then filtered through a 7 μm filter paper (Hana BioLab, Hanam, Republic of Korea). The ethanol extract was combined with γ-CD (Wacker, Ann Arbor, MI, USA) in a 1:2 molar ratio and stirred using a vortex mixer at 80 °C for 30 min as previously described [21], forming the inclusion complex of MPE and γ-CD. After incorporating 5% lecithin (Shankar Soya Concepts, Indore, India) and 10% sodium casein (Lactoprot, Kaltenkirchen, Germany) into the inclusion complexes, the mixture was homogenized at 50 °C for 30 min using a homogenizer (Nissei, Nagano-ken, Japan). The sample solution was then stabilized at 4 °C for 24 h and freeze-dried at −40 °C for 96 h using a freeze-dryer (Lyoph Pride 20, Ilshin Bio Base, Seoul, Republic of Korea). The resultant MPE-loaded liposomes were dissolved in DMSO (Sigma-Aldrich, St. Louis, MO, USA) to create a stock solution (50 mg/mL).

### 4.3. Determination of α-MG Content in MPE and MPE-Loaded Liposomes

The α-MG content in the MPE and MPE-loaded liposomes was measured using the Agilent 1260 InfinityII Series HPLC-UVD (Agilent Technologies, Santa Clara, CA, USA) and a Waters Sunfire C18 column (4.6 × 250 mm, 5 µm size) (Milford, MA, USA). The mobile phases consisted of 0.1% phosphoric acid in water (A) and methanol (B). The elution followed a gradient elution: 80% B for 0–8 min, 80% B to 100% B for 11 min, constant at 100% B for 6 min, reducing from 100% B to 80% B in 1 min, and then constant at 80% B for 4 min. The flow rate of the samples was 1.0 mL/min at 25 °C. DAD detector was set at a wavelength of 320 nm with an injection volume of 5 μL for each sample and standard. α-MG (>98% purity, Phytolab, Vestenbergsgreuth, Germany) was prepared by dissolving 5.0 mg of α-MG in 0.5 mL of DMSO and adjusted to 25 mL with methanol. Various concentrations of this standard solution were diluted in methanol with 2% DMSO for the calibration curve. Each sample (20 mg) was placed in volumetric flasks filled with methanol containing 2% DMSO and sonicated for 30 min. The α-MG contents in MPE and MPE-loaded liposomes were determined using these calibration curves. The limit of quantification and limit of detection was measured as previously described [30].

### 4.4. Bacterial Strains

Eight *Staphylococcus* species and two Gram-negative bacteria, *E. coli* and *P. aeruginosa*, were sourced from the Korean Collection for Type cultures (KCTC, Jeongeup-si, Republic of Korea) and the American Type Culture Collection (ATCC; Manassas, VA, USA) (Table 1). Clinical isolates from skin diseases of companion dogs (*n* = 206) and cats (*n* = 71) were obtained from the Animal and Plant Quarantine Agency (Gimcheon, Korea): *E. coli* (*n* = 20), *P. aeruginosa* (*n* = 16), *S. aureus* (*n* = 19), *S. epidermidis* (*n* = 27), *S. felis* (*n* = 51), *S. pseudintermedius* (*n* = 80), and *S. schleiferi* (*n* = 64). These clinical isolates were the same as those used in a previous study [16].

### 4.5. Antimicrobial Susceptibility Testing

The MICs were determined using broth microdilution or agar dilution methods according to the Clinical Laboratory Standards Institute guidelines [31]. Serial two-fold dilutions of MPE-loaded liposomes were prepared in Mueller–Hinton broth (Difco, Detroit, MI, USA) or on Mueller–Hinton agar. The MICs of α-MG (Combi-blocks, San Diego, CA, USA) against reference bacterial strains were determined using broth microdilution method as a positive control. A bacterial suspension of 5 × 10^5^ CFUs/mL was inoculated into the media containing MPE-loaded liposomes and incubated at 37 °C for 20 h. The MBCs were determined by plating the broth dilution samples onto Mueller–Hinton agar plates.

### 4.6. Cell Culture and Cytotoxicity Assay

CPEK cells (CELLnTEC Advanced Cell System, Bern, Switzerland) were utilized in this study. Cells were seeded in a 96-well plate at a density of 8.0 × 10^3^ cells/well and treated with various concentrations (28–660 µg/mL) of MPE-loaded liposomes for 24 h. Cell viability was assessed using a 3-(4,5-dimethylthiazol-2yl)-2,5-diphenyltetrazolium bromide (Sigma-Aldrich). MTT dye was added to each well and incubated for 4 h. Cell culture medium was removed and the formazan crystals generated by oxidation of the MTT dye by mitochondria were dissolved in 0.04 N HCl in isopropanol. The absorbance was measured at 570 nm. The cell survival ratio was expressed as a percentage of the control viability. The experiments were conducted in three independent experiments.

### 4.7. Transmission Electron Microscopy Analysis

The MPE-loaded liposomes (3.75 mg in 1 mL water) were applied to a copper grid (Electron Microscopy Sciences, Hatfield, PA, USA). Excess water was blotted from the specimen using filter paper. The samples were then stained with 2% uranium acetate and visualized under a transmission electron microscope (JEM-1400; Jeol, Tokyo, Japan).

### 4.8. Mouse Skin Infection and Application of MPE-Loaded Liposomes

Six-week-old female BALB/c mice were obtained from Hyochang Science (Daegu, Republic of Korea). Mice were maintained in a flow chamber at 22 ± 2 °C and relative humidity of 55% ± 5%. *S. pseudintermedius* ATCC 49051 was cultured overnight in trypticase soy broth (Difco, Detroit, MI, USA), then diluted 1:100 and grown to an optical density at 600 nm (OD_600_) of 1.0. Bacteria were resuspended in PBS at a concentration of approximately 1.0 × 10^10^ CFUs/mL. After the backs of the mice were shaved with a sterile blade, the skin was sterilized using 70% ethanol and dried. Subsequently, the skin was stripped 20–25 times using elastic bandage tape to disrupt the cutaneous barrier. Bacteria (1.1~1.3 × 10^9^ CFUs/100 µL) were inoculated onto the skin lesions. MPE-loaded liposomes (550 µg, containing 10 µg α-MG) were applied to the lesions every 12 h for 6 days. As positive and negative controls, α-MG (10 µg) dissolved in DMSO and 1% DMSO in PBS were similarly applied for 6 days at the same intervals of the treatment group, respectively. CFUs in the skin lesions were assessed on mannitol salt agar plates (Difco) on days 1, 3, and 7. The size of reddish skin lesions was measured using the Image J software (https://imagej.net/ij/, accessed on 15 April 2024). All animal experiments adhered to the guidelines of the Institutional Animal Care and Use Committee of Kyungpook National University (KNU-2022-0327).

### 4.9. Histological Analysis

Mice were euthanized on day 7, and skin lesions were excised. Tissue samples were fixed in 10% paraformaldehyde and embedded in a paraffin block. The blocks were then serially sectioned into 3 μm-thick slices and stained with hematoxylin and eosin (H&E).

### 4.10. Statistical Analysis

Statistical analyses were performed using GraphPad Prism 5.0 (San Diego, CA, USA). A one-way analysis of variance with Dunnett’s post-hoc analysis and Student’s *t*-tests were performed. Differences with *p* < 0.05 were considered statistically significant.

## 5. Conclusions

This study demonstrates the antibacterial and wound healing effects of liposome formulation of MPE on superficial skin lesions caused by *S. pseudintermedius*. MPE-loaded liposomes emerge as a promising candidate for the development of topical therapeutics for superficial pyoderma in companion animals.

## Figures and Tables

**Table 1:** In vitro antibacterial activity of mangosteen pericarp extract-loaded liposomes against reference bacterial strains.

Bacteria	MIC (µg/mL) of α-MG *	MIC (µg/mL) of MPE-Loaded Liposomes		MBC (µg/mL) of MPE-Loaded Liposomes
		Broth Microdilution	Agar Dilution	
*S. aureus* ATCC 29213	2	220 (4) **	880 (16)	220 (4)
*S. carprae* KCTC 3583	2	110 (2)	220 (4)	110 (2)
*S. epidermidis* ATCC 12228	2	110 (2)	110 (2)	110 (2)
*S. felis* ATCC 49168	2	55(1)	110 (2)	55 (1)
*S. intermedius* KCTC 3344	2	110 (2)	220 (4)	110 (2)
*S. pseudintermedius* ATCC 49051	2	55 (1)	110 (2)	55 (1)
*S. saprophyticus* KCTC 3345	1	55 (1)	110 (2)	55 (1)
*S. schleiferi* ATCC 43808	2	55 (1)	55 (1)	55 (1)
*E. coli* ATCC 25922	>64	>3520 (>64)	>3520 (>64)	>3520 (>64)
*P. aeruginosa* ATCC 27853	>64	>3520 (>64)	>3520 (>64)	>3520 (>64)

MIC, minimum inhibitory concentration; MBC, minimum bactericidal concentration. * The MICs of α-MG were determined using the broth microdilution method. ** Values in parenthesis are MICs and MBCs of α-MG in MPE-loaded liposomes.

**Table 2:** In vitro antibacterial activity of mangosteen pericarp extract-loaded liposomes against clinical isolates of *Staphylococcus* species and Gram-negative bacteria.

Bacterial Isolates (No.)	No of Isolates with the Following MIC * (µg/mL)							MIC_50_ (µg/mL)	MIC_90_ (µg/mL)
	28 (0.5) **	55 (1)	110 (2)	220 (4)	440 (8)	880 (16)	>3520 (>64)		
*S. aureus* (19)					6	13		880 (16)	880 (16)
*S. epidermidis* (27)			1	15	9	2		220 (4)	440 (8)
*S. felis* (51)			10	41				220 (4)	220 (4)
*S. pseudintermedius* (80)	1	2	73	4				110 (2)	110 (2)
*S. schleiferi* (64)			23	41				220 (4)	220 (4)
*E. coli* (20)							20	>3520 (>64)	>3520 (>64)
*P. aeruginosa* (16)							16	>3520 (>64)	>3520 (>64)

MIC, minimum inhibitory concentration; MIC_50_, MIC of 50% of the isolates; MIC_90_, MIC of 90% of the isolates. * The MICs of MPE-loaded liposomes were tested using the agar dilution method. ** Values in parenthesis are MICs of α-MG in MPE-loaded liposomes.

## Data Availability

The authors confirm that the data supporting the findings of this study are available within the article.
